# Cell size heterogeneity controls crystallization of the developing fruit fly wing

**Published:** 2026-10-23

**Authors:** Kartik Chhajed, Franz S. Gruber, Raphaël Etournay, Natalie A. Dye, Frank Jülicher, Marko Popović

## Abstract

A fundamental question in biology is to understand how patterns and shapes emerge from the collective interplay of large numbers of cells. Cells forming two-dimensional epithelial tissues behave as active materials that undergo remodeling and spontaneous shape changes. Focusing on the fly wing as a model system, we find that the cellular packing in the wing epithelium transitions from a disordered packing to an ordered, crystalline packing. While previous studies propose a role of tissue shear flow in establishing the ordered cell packing in the fly wing, we reveal a role of cell size heterogeneity. Indeed, we find that even if tissue shear have been inhibited, cell packings in the fruit fly wing epithelium transition from disordered to an ordered packing. We propose that the transition is controlled by the cell size heterogeneity, which is quantified by the cell size polydispersity. To explore the role of cell size polydispersity in controlling cellular packings, we implement polydispersity in a vertex model of epithelial tissues. Through numerical simulations of this model, we show that there is a critical value of cell size polydispersity above which cellular packings are disordered and below which they form a crystalline packing. By analyzing experimental data, we find that cell size polydispersity decreases during fly wing development. The observed dynamics of tissue crystallisation is consistent with the slow ordering kinetics we observe in the vertex model. Therefore, although tissue shear does not control the transition, it significantly enhances the rate of tissue-scale ordering by facilitating alignment of locally ordered crystallites. Our results identify cell size heterogeneity as a control parameter, in both the vertex model and the fruit fly wing epithelium, controlling the transition between ordered and disordered cellular packings.

